# Correction to: Consistent methods for fat free mass, creatinine clearance, and glomerular filtration rate to describe renal function from neonates to adults

**DOI:** 10.1002/psp4.13081

**Published:** 2023-11-10

O'Hanlon, CJ, Holford, N, Sumpter, A, Al‐Sallami, HS. Consistent methods for fat‐free mass, creatinine clearance, and glomerular filtration rate to describe renal function from neonates to adults. *CPT Pharmacometrics Syst Pharmacol*. 2023; 12: 401–412. doi:10.1002/psp4.12924

In the published version of O'Hanlon, CJ, et al. (2023) we would like to correct the following.

Equation 7 should read:

FFMIN=FMAT×FMAT_MAX

The associated Equation 7 text currently reads:

“The baseline, FFMIN, is obtained from FMAT_PRE, a parameter describing FRFFM in a 24‐week premature neonate, and FMAT_MAX, the asymptotic estimate of FFMadult (Equation 7).”

But should read:

“The baseline, FFMIN, is obtained from FFMAT, the fraction of FMAT_MAX describing the nadir of FRFFM and FMAT_MAX, the asymptotic estimate of FFMadult (Equation 7).”

Equation 8 should read:

$$\text{FFNEO}=\left(\text{FMAT}\_\mathrm{PRE}-\text{FFMIN}\right)\times e^{-\frac{\log\left(2\right)}{\mathrm{TFF}\_\mathrm{PRE}}\times \left(\mathrm{PMA}-\frac{24}{52}\right)}$$

Figure 1 should be:

**FIGURE 1:**
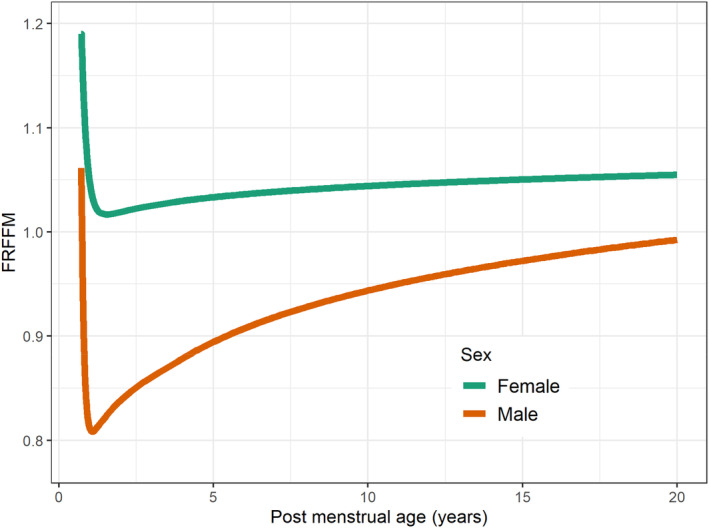
Predicted fraction of adult FFM (FRFFM) in males and females from neonates to young adults based on postmenstrual age, sex, total body mass, and height covariates in the pooled data set. FRFFM rises approaching adult fat free mass values with an asymptote of 1 in males, but with a higher asymptote of 1.06 in females. Parameter values are shown in Table 1.

We apologize for these errors.

